# Comparison of rumen bacteria distribution in original rumen digesta, rumen liquid and solid fractions in lactating Holstein cows

**DOI:** 10.1186/s40104-017-0142-z

**Published:** 2017-02-01

**Authors:** Shoukun Ji, Hongtao Zhang, Hui Yan, Arash Azarfar, Haitao Shi, Gibson Alugongo, Shengli Li, Zhijun Cao, Yajing Wang

**Affiliations:** 1 State Key Laboratory of Animal Nutrition, Beijing Engineering Technology Research Center of Raw Milk Quality and Safety Control, China Agricultural University, Beijing, 100193 China; 2 Faculty of Agriculture, Lorestan University, PO Box 465, Khorramabad, Iran

**Keywords:** Bacteria biomarker, Rumen bacteria diversity, Rumen content fraction

## Abstract

**Background:**

Original rumen digesta, rumen liquid and solid fractions have been frequently used to assess the rumen bacterial community. However, bacterial profiles in rumen original digesta, liquid and solid fractions vary from each other and need to be better established.

**Methods:**

To compare bacterial profiles in each fraction, samples of rumen digesta from six cows fed either a high fiber diet (HFD) or a high energy diet (HED) were collected via rumen fistulas. Rumen digesta was then squeezed through four layers of cheesecloth to separate liquid and solid fractions. The bacterial profiles of rumen original digesta, liquid and solid fractions were analyzed with High-throughput sequencing technique.

**Results:**

Rumen bacterial diversity was mainly affected by diet and individual cow (*P* > 0.05) rather than rumen fraction. Bias distributed bacteria were observed in solid and liquid fractions of rumen content using Venn diagram and LEfSe analysis. Fifteen out of 16 detected biomarkers (using LEfSe analysis) were found in liquid fraction, and these 15 biomarkers contributed the most to the bacterial differences among rumen content fractions.

**Conclusions:**

Similar results were found when using samples of original rumen digesta, rumen liquid or solid fractions to assess diversity of rumen bacteria; however, more attention should be draw onto bias distributed bacteria in different ruminal fractions, especially when liquid fraction has been used as a representative sample for rumen bacterial study.

**Electronic supplementary material:**

The online version of this article (doi:10.1186/s40104-017-0142-z) contains supplementary material, which is available to authorized users.

## Background

The bovine rumen harbors a diverse population of microorganisms that convert ingested plant biomass into microbial protein and volatile fatty acids, and their fermentation end-products provide the host with essential nutrients for metabolism. Rumen microbes, therefore, play a key role in the productivity and health of ruminants [1].

Collection and sampling of ruminal content are important in both scientific research and diagnosis of diseases in ruminants [2]. Ruminal microbial diversity has been investigated in numbers of studies using different ruminal fractions including original rumen digesta, rumen liquid or solid fractions [3–8]. It has been demonstrated that rumen sampling methods and/or sampling pre-treatments could affect on the results of rumen microbial community [9, 10]. The relationships among microbial communities in different fractions of rumen content have been studied previously [11, 12]; however, it is still a controversial topic and deserves further investigations. In late studies, development of high-throughput sequencing techniques have allowed subtle effects on microbial community components to be detected as changes in relative numbers of bacterial community [1]. The objective of this study was to assess the differences and similarities of bacterial community in original rumen digesta, rumen liquid and solid fractions using a high-throughput sequencing technique.

## Methods

### Animals and sampling

Six ruminally fistulated lactating Holstein cows were housed in a free stall pen at the Zhongdi Dairy Research Center (Beijing, China) and were cared for according to the practices outlined in the Guide for the Care and Use of Agriculture Animals in Agriculture Research and Teaching (FASS, 2010).

Cows were randomly assigned to two groups with three cows in each group and individually fed by Roughage Intake Control System (RIC, Insentec B.V, Netherland). One group of cows were fed with a high fiber containing diet (HFD group) and the other group was fed with a high energy containing diet (HED group) (Table 1). After a 14-days adaptation period to the experimental diets, approximately 500 g of original rumen digesta of each cow was collected 5 h after morning feeding via rumen fistulas from the middle part of the ventral sac. After the original rumen digesta sampled, the solid and liquid fractions were obtained by squeezing the original digesta through four layers of sterile cheesecloth. All samples were snap-frozen in liquid nitrogen and were stored at −80 °C until DNA extraction.

**Table 1:** Ingredients and chemical composition of the experimental diets (as dry matter basis)

Items^a^	High energy diet (HED)	High fiber diet (HFD)
Ingredients, kg		
Corn silage	4.51	4.86
Alfafa hay	2.38	1.39
Oat hay	─	2.66
Extruded soybean	0.35	0.27
Flaked corn	3.26	1.32
Corn	1.72	2.87
DDGS	1.79	1.28
Salt	0.08	0.07
Sodium bicarbonate	0.29	0.29
Cottonseed	1.36	0.90
Soybean meal	2.12	1.82
Beet pulp	1.00	0.43
Additives	2.04	1.34
Contents, %		
DM as fed	56.4	53.5
Crude protein	17.98	17.00
NE_L_, MCal/kg^b^	1.81	1.67
Fat	5.78	4.43
NDF	32.84	37.81
ADF	17.86	20.53
NFC	40.02	38.35
Ca	0.80	0.80
P	0.39	0.36

^a^ *DDGS* dried distillers grains with solubles, *DM* dry matter, *NE*_*L*_ net energy requirement for lactation, *NDF* neutral detergent fiber, *ADF* acid detergent fiber, *NFC* nonfiber carbohydrates, *Ca* calcium, *P* phosphorus

^b^Calculated using equations from NRC (2001)

### DNA isolation

Genomic DNA (gDNA) was extracted from 1 g of original or solid fraction, and from 1 mL of ruminal liquid with Qiagen DNA Extraction Kit™ (Qiagen, Hilden, Germany) using a repeated bead beating method followed by phenol-chloroform extraction according to the manufacturer’s protocol. The DNA was re-suspended after being precipitated with ethanol. The quality of extracted DNA was assessed based on the absorbance ratios of 260/280 nm and 260/230 nm using a NanoDrop ND-1000 Spectrophotometer (NanoDrop Technologies, Wilmington, DE, USA). The values for A260/A280 ratio in the present study were ranged from 1.8 to 2.0.

### PCR amplification and purification

For illumina MiSeq sequencing, bacterial 16S rRNA gene were amplified using primers covering the V3 region (343 F, 5′-GATCCTACGGGAGGCAGCA-3′ and 534R, 5′-GCTTACCGCGGCTGCTGGC-3′) with barcodes. All PCR reactions were carried out in 30 μL reaction mixtures with 15 μL of Phusion High-Fidelity PCR Master Mix (New England Biolabs), 0.2 μL of both forward and reverse primers and 10 ng of template DNA. PCR amplification was carried out according to the following protocol: initial denaturation for 5 min at 95 °C, followed by 25 cycles of denaturation at 95 °C for 1 min, annealing at 50 °C for 1 min, and elongation at 72 °C for 1 min, with a final elongation step at 72 °C for 7 min. To qualify and quantify PCR products, the same volume of 1 × loading buffer (containing SYBR green) mixed with PCR products and electrophoresis on 2% agarose gel. Samples with bright main strip between 200 and 210 bp were then chosen for further analysis. PCR products from samples for sequencing in the same MiSeq run were pooled at equal molality. The pooled mixture was purified with a QIAquick PCR Purification Kit (Qiagen, Hilden, Germany) and re-quantified with Agilent DNA 1000 Kit (Agilent Technologies Inc.).

### Sequencing with high-throughput sequencing technique

Sequencing libraries were generated using NEBNext ultra DNA sample preparation kit (NEB, USA), following standard Illumina sample-preparation protocol. The quality of library was assessed on the Qubit 2.0 Fluorometer (Life technologies, Grand Island, NY, USA) and Agilent Bioanalyzer 2100 system (Agilent Technologies, Palo Alto, Calif.). The library was sequenced on illumina MiSeq platform and ~250 bp to ~300 bp paired-end reads were generated.

### Quality control of raw data and data processing

Controlling the quality of raw data was done by FastQC (version 0.11.3). Reads with quality score higher than 30 were retained for further analysis. Paired-end reads from the original DNA fragments were merged using FLASH (version 1.2.7) [13]. Paired-end reads was assigned to each sample according to the unique barcodes. Concatenated sequences were detected using USEARCH (v6.1), and subsequently filtered out. Sequences analyses were performed using QIIME pipeline (version 1.5.0) [14]. Generated sequences were distributed into different samples based on barcodes, and the OTUs were defined by clustering sequences together with a 97% identity cut-off at UCLUST software [15] after removing the barcode. The RDP classifier [16] was used for taxonomic classification of generated OTUs [17]. To ensure the comparability of the species diversity between the samples, standardized OTU documents were used to analyze the species and diversity indexes. The threshold for the number of standardized sequences was set at 150,000 sequences.

### Data analysis

Alpha diversity indices were calculated using QIIME pipeline (version 1.5.0) [14]. Beta diversity indices between samples were determined based on Bray-Curtis metric, relationship network of each fraction was calculated using Pearson correlation. LEfSe (LDA Effect Size) analysis was performed online (https://huttenhower.sph.harvard.edu/galaxy) to find differentially abundant taxa (biomarkers) with *P*- value higher than 0.05 and LDA score higher than 2. ANOSIM analysis was performed with R software (version 3.1.2). Comparisons of bacterial abundance in different experimental groups were performed using Wilcoxon or Kruskal-Wallis test with R software (version 3.1.2).

## Results

### Bacterial diversities and community composition in different rumen fractions

Six ruminally fistulated Holstein dairy cows were grouped by two different diets containing either high level of fiber or high level of energy. Rumen contents were collected and separated to liquid and solid fractions. After sequencing on the Illumina MiSeq platform, 4,650,173 quality reads were generated, and 23,896 OTUs in total in all samples were detected with an average of 258,343 ± 26,890 reads and 5,003 ± 678 OTUs in each sample.

Within each dietary treatment, the number of OTUs and Chao1 index between different fractions of rumen samples were similar (*P* > 0.05); however, the number of OTUs and Chao1 index in rumen liquid fraction was higher in HFD group than that of HED group (*P* < 0.05; Additional file 1: Figure S1a and b). Simpson index has no difference among fractions within and between dietary treatments (*P* > 0.05; Additional file 1: Figure S1c).

**Fig. 1:**
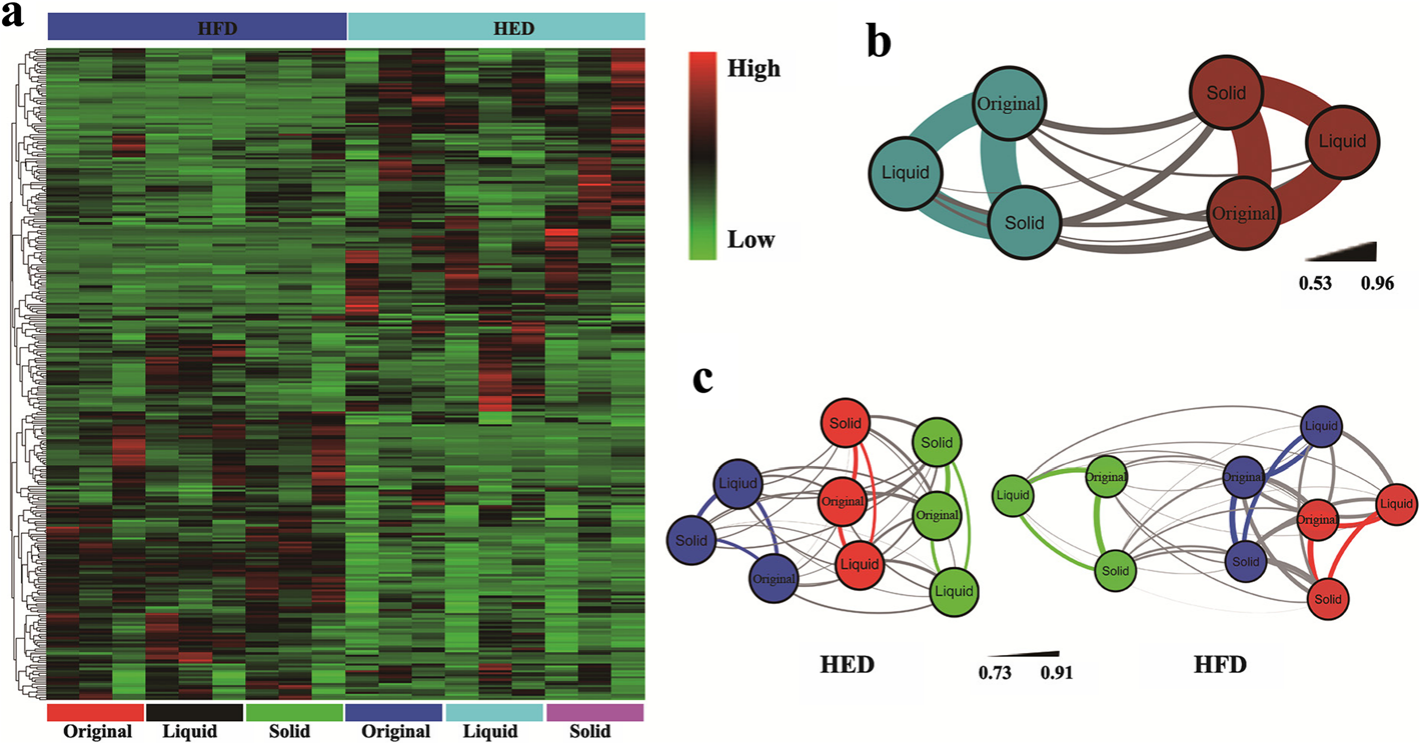
Comparison of bacterial community in different fractions of rumen content based on OTUs. **a**, Heatmap of top 300 OTUs. **b**, Pearson correlation similarity of fractions, grayblue nodes represent HFD and darkred nodes represent HED, the thickness of lines indicates the Pearson correlation similarity. **c**, Pearson correlation similarity of different fractions of rumen content in HED group (*right*) and HFD group (*left*), nodes in same color represent samples obtained from the same cow, the thickness of lines indicates the Pearson correlation similarity

The microbial diversity difference was displayed by heatmap, the similarity index with Pearson correlation, and ANOSIM analysis with Bray-Curtis similarity (Fig. 1; Additional file 1: Figure S2). We found that bacteria communities were clustered mainly by diets (Fig. 1a, b; Additional file 1: Figure S2a) and individual cows (Fig. 1c; Additional file 1: Figure S2b).

Venn plot was used to illustrate the distribution of bacteria in different ruminal fractions. After filtering out the rare OTUs (defined as OUTs that only appear in one sample in each group), we identified 2,022 OTUs appeared in ruminal fractions. More unique OTUs (appeared in one fraction but not appeared in others) were detected in HFD group compared with that in HED group (Additional file 1: Figure S3a). Rumen original digesta, solid and liquid fractions shared 53% of detected OTUs, but unique OTUs was also found in different fractions although they were evenly distributed among the rumen fractions (Additional file 1: Figure S3b).

### Rumen bacterial taxa change in original and fractional rumen digesta

**Fig. 2:**
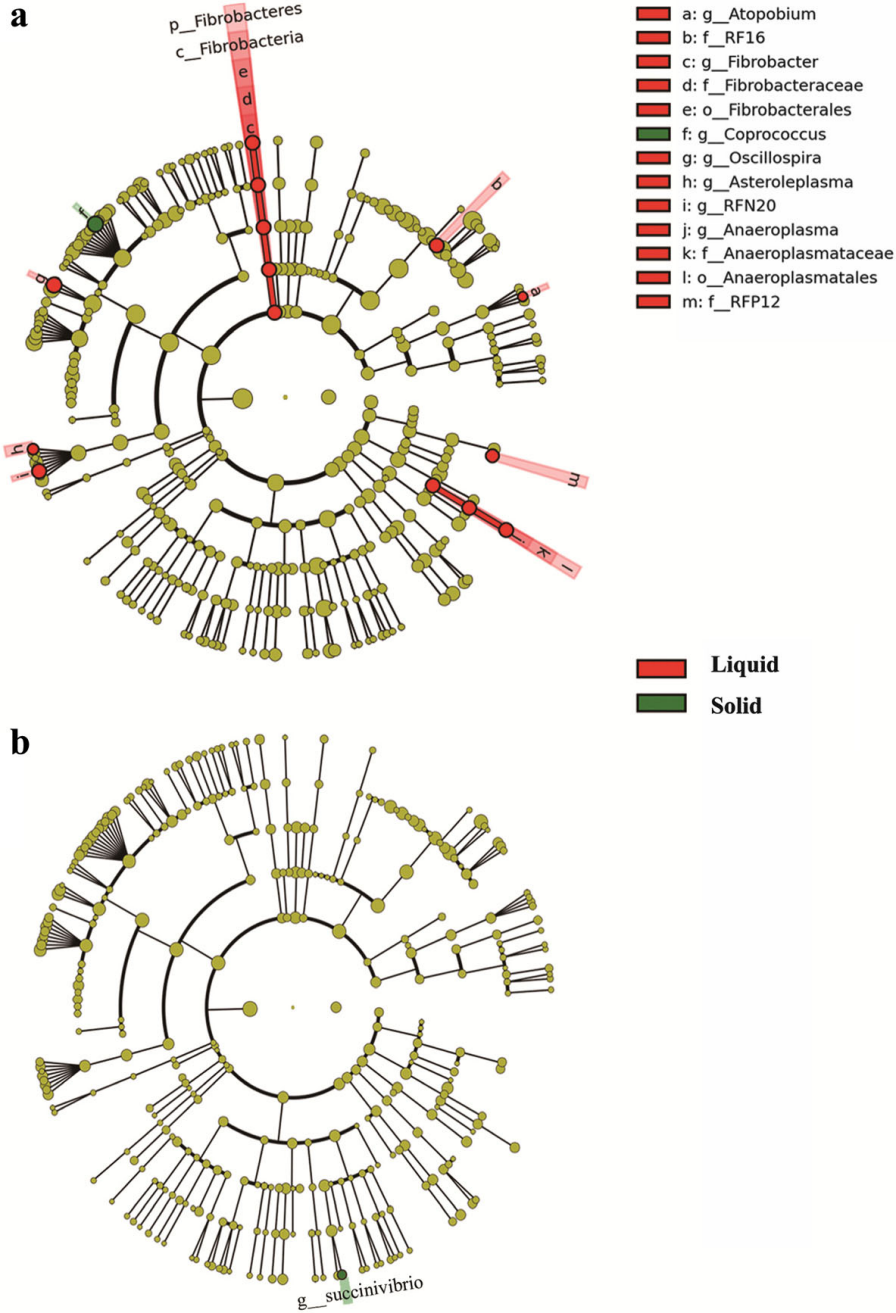
Ruminal bacteria change in different fractions at genus level. LEfSe cladograms demonstrating taxonomic differences among different fractions in HFD group (**a**) and HED group (**b**) respectively, LDA scores above 2 and *P* value smaller than 0.05 were shown. LEfSe: linear discriminant analysis (LDA) effect size

In total, 22 phyla, 38 classes, 62 orders, 96 families, 127 genera were detected regardless of fractional and dietary treatments (Fig. 2). To identify the taxon distributions in different fractions, LEfSe analysis was performed and biomarkers of liquid and/or solid fractions were found in both HFD and HED groups (Fig. 2). Fifteen taxa were found as biomarker in HFD group (Fig. 2a; Additional file 1: Figure S4a), while only one taxon was detected as biomarker in HED group (Fig. 2b; Additional file 1: Figure S4b).

Within HFD group, 14 taxa increased in liquid fraction and one taxon increased in solid fraction (Fig. 2a; Additional file 1: Figure S5). Among the changed taxa, genus *Coprococcus* and *Oscillospira* were found to be predominant bacteria (appeared in all samples and relative abundance ≥ 1% in at least one sample; Additional file 1: Figure S6) in both rumen solid and liquid fractions in HFD group, respectively. Within HFD group, relative abundance of genus *Succinivibirio* among ruminal fractions were found different (Fig. 2b) with the highest abundance found in solid fraction (*P* < 0.05; Additional file 1: Figure S5).

## Discussion

### Influence of rumen content fractions on bacterial diversity

Rumen, harboring large number of inhabiting microbes (approximately 10^11^ bacterial cells per g of rumen content) which play important roles in providing necessary nutrients (such as proteins and energy yielding substrates) to the host animal [18, 19]. Many factors such as age [4], diet [11, 19], and animal individual [20] affect rumen microbial community. Original rumen digesta, liquid fraction and solid fraction samples have been frequently used to assess rumen microbes [1, 4]; however, the fractions used may also cause biased observation in rumen microbial studies [1, 20]. In present study, we examined the differences and similarities of bacterial communities in different rumen fractions from lactating dairy cows fed with either HFD or HED using high throughput sequencing technology.

Many researchers proved that diet is one of the main factors that affect rumen microbial diversity, and that the nutritional plane and/or feed ingredients have great impacts on rumen microbial communities [3, 21–25], which may due to bacteria preferring particular metabolic substrates and rumen environment [26]. This was supported by our findings that HFD- and HED-fed cows harbored different bacterial communities (Fig. 1a; Additional file 1: Figure S3a), which was illustrated by different α-diversity indices (Additional file 1: Figure S1) and different clusters (Fig. 1b; Additional file 1: Figure S2a) between dietary treatment groups.

Regardless the dietary effect, bacterial community of each individual cow was quite unique (Fig. 1c). A clear grouping resulted by individual cows (Additional file 1: Figure S2b) indicating that animal individual had its own distinct microbiota [27]. This finding was also supported by a previous study in which cows were switched from a high forage diet to a high concentrate diet (during acidosis and after recovery) and the bacterial populations exhibited a low taxonomic variability as less than 5% of total identified OTUs differed [11].

Between different ruminal content fractions, the diversity difference was not observed (Fig. 1b and c; Additional file 1: Figure S1; Figure S2c), while different bacteria distribution was detected (Fig. 1a; Additional file 1: Figure S3b) indicating that each fraction had some unique bacteria.

### Influence of ruminal fractions on bacterial taxon distribution

Different bacteria prefer particular metabolic substrates and rumen environment and thus might be distributed differently in disparate phases [26]. In the current study, results of LEfSe illustrated that distribution difference of rumen bacteria in different fractions can be observed in both HFD group (Fig. 2a) and HED group (Fig. 2b). Biomarkers of liquid and solid fractions were detected (Fig. 2), and bacterial community of original digesta were largely likely displaying an intermediate state of liquid and solid fractions (Additional file 1: Figure S5). This verifies the common theory that bacterial community in original digesta represents the real rumen bacterial community the best.

Crucially, we found that liquid fraction contributed most to the bacteria difference in different fractions of HFD group (Fig. 2; Additional file 1: Figure S5). Usually, for studying rumen microbial diversity, original rumen digesta sampling through a fistula or from slaughtered animals was described the best method to have a representative sample [27]. Alternatively, rumen liquid collected via a stomach tube has become a routinely used method for rumen sample collection because of its easy achievement [1, 28, 29]. A previous study showed significant differences in bacterial communities between rumen solid and liquid content [21]. In current study, liquid fraction had similar bacterial diversity with original rumen digesta and solid fraction, but particular bacteria abundance (including predominant bacteria) in liquid fraction differed with original content and solid fraction (Additional file 1: Figure S5; Figure S6). The biased distribution of bacteria should be taken into consideration when samples of liquid fraction have been used to assess rumen bacterial community.

## Conclusions

This study investigated and compared rumen bacteria community of original rumen digesta, liquid and solid fractions from cows fed with HFD and HED. Rumen bacteria diversity was mainly affected by diet and cow individuals rather than by rumen fractions. Bias distributed bacteria were observed in different fractions of rumen content. Liquid fraction contributed most to bacterial differences among rumen content fractions of HFD group. Results indicated that using different fractions to assess rumen bacterial diversity will generate similar results; however, more attention should be paid to bias distributed bacteria in different fractions, especially when liquid fraction is used as a representative sample.

## Additional files

Additional file 1: Figure S1. Microbial diversity in different fractions of rumen content. a, the OTU numbers in original, solid or liquid fraction samples. b, Chao1 index in original, solid or liquid fraction samples. c, Simpson index based on OTUs in original, solid, and liquid fraction samples. HFD: High fiber diet; HED: High energy diet. Data are presented as Mean ± SD. **Figure S2.** Analysis of similarity (ANOSIM) in different groups. ANOSIM results are presented with box plot when bacteria communities are grouped by diet (a), cows (b), and ruminal content fractions (c) using Bray-Curtis metric based on OTUs. **Figure S3.** Venn plot for shared OTUs. a, OTUs in HFD and HED. b, OTUs in original, solid and liquid fractions. **Figure S4.** Ruminal bacteria change in different fractions of rumen content at genera level. LEfSe histogram demonstrating taxonomic differences among different fractions in HFD group (a) and HED group (b) respectively, LDA scores above 2 and P value smaller than 0.05 were shown. LEfSe: linear discriminant analysis (LDA) effect size. **Figure S5.** Influence of rumen fractions on biomarker taxa abundance. p_: phylum; c_: class; o_: order; f_: family; g_: genus. Data was presented as Mean ± SD. **Figure S6.** Predominant rumen bacteria at genera level. a, predominant genera higher than 1% in proportion in all samples. b, distribution of predominant genera in each fractions. (DOC 1371 kb)
Additional file 2: OTUs distribution in each sample. (XLSX 2056 kb)

## Abbreviations

ADF: Acid detergent fiber
Ca: Calcium
DDGS: Dried distillers grains with soluble
DM: Dry matter
HED: High energy diet
HFD: High fiber diet
NDF: Neutral detergent fiber
NE_L_: Net energy requirement for lactation
NFC: Nonfiber carbohydrates
OTUs: Operational Taxonomic Units
P: Phosphorus
