# Supplementary material for: Comparison of rumen bacteria distribution in original rumen digesta, rumen liquid and solid fractions in lactating Holstein cows
Source: J Anim Sci Biotechnol. 2017 Feb 1;8:16. doi: 10.1186/s40104-017-0142-z (PMC5286850; doi:10.1186/s40104-017-0142-z)
Supplement: Additional file 1: Figure S1. — Microbial diversity in different fractions of rumen content. a, the OTU numbers in original, solid or liquid fraction samples. b, Chao1 index in original, solid or liquid fraction samples. c, Simpson index based on OTUs in original, solid, and liquid fraction samples. HFD: High fiber diet; HED: High energy diet. Data are presented as Mean ± SD. Figure S2. Analysis of similarity (ANOSIM) in different groups. ANOSIM results are presented with box plot when bacteria communities are grouped by diet (a), cows (b), and ruminal content fractions (c) using Bray-Curtis metric based on OTUs. Figure S3. Venn plot for shared OTUs. a, OTUs in HFD and HED. b, OTUs in original, solid and liquid fractions. Figure S4. Ruminal bacteria change in different fractions of rumen content at genera level. LEfSe histogram demonstrating taxonomic differences among different fractions in HFD group (a) and HED group (b) respectively, LDA scores above 2 and P value smaller than 0.05 were shown. LEfSe: linear discriminant analysis (LDA) effect size. Figure S5. Influence of rumen fractions on biomarker taxa abundance. p_: phylum; c_: class; o_: order; f_: family; g_: genus. Data was presented as Mean ± SD. Figure S6. Predominant rumen bacteria at genera level. a, predominant genera higher than 1% in proportion in all samples. b, distribution of predominant genera in each fractions. (DOC 1371 kb) [file 40104_2017_142_MOESM1_ESM.doc]

Additional file 1

Figure S1. Microbial diversity in different fractions of rumen content. a, the OTU numbers in original, solid or liquid fraction samples. b, Chao1 index in original, solid or liquid fraction samples. c, Simpson index based on OTUs in original, solid, and liquid fraction samples. HFD: High fiber diet; HED: High energy diet. Data are presented as Mean±SD.

Figure S2. Analysis of similarity (ANOSIM) in different groups. ANOSIM results are presented with box plot when bacteria communities are grouped by diet (a), cows (b), and ruminal content fractions (c) using Bray-Curtis metric based on OTUs.

Figure S3. Venn plot for shared OTUs. a, OTUs in HFD and HED. b, OTUs in original, solid and liquid fractions.

Figure S4. Ruminal bacteria change in different fractions of rumen content at genera level. LEfSe histogram demonstrating taxonomic differences among different fractions in HFD group (a) and HED group (b) respectively, LDA scores above 2 and P value smaller than 0.05 were shown. LEfSe: linear discriminant analysis (LDA) effect size.

Figure S5. Influence of rumen fractions on biomarker taxa abundance. p_: phylum; c_: class; o_: order; f_: family; g_: genus. Data was presented as Mean ± SD.

Figure S6. Predominant rumen bacteria at genera level. a, predominant genera higher than 1% in proportion in all samples. b, distribution of predominant genera in each fractions.


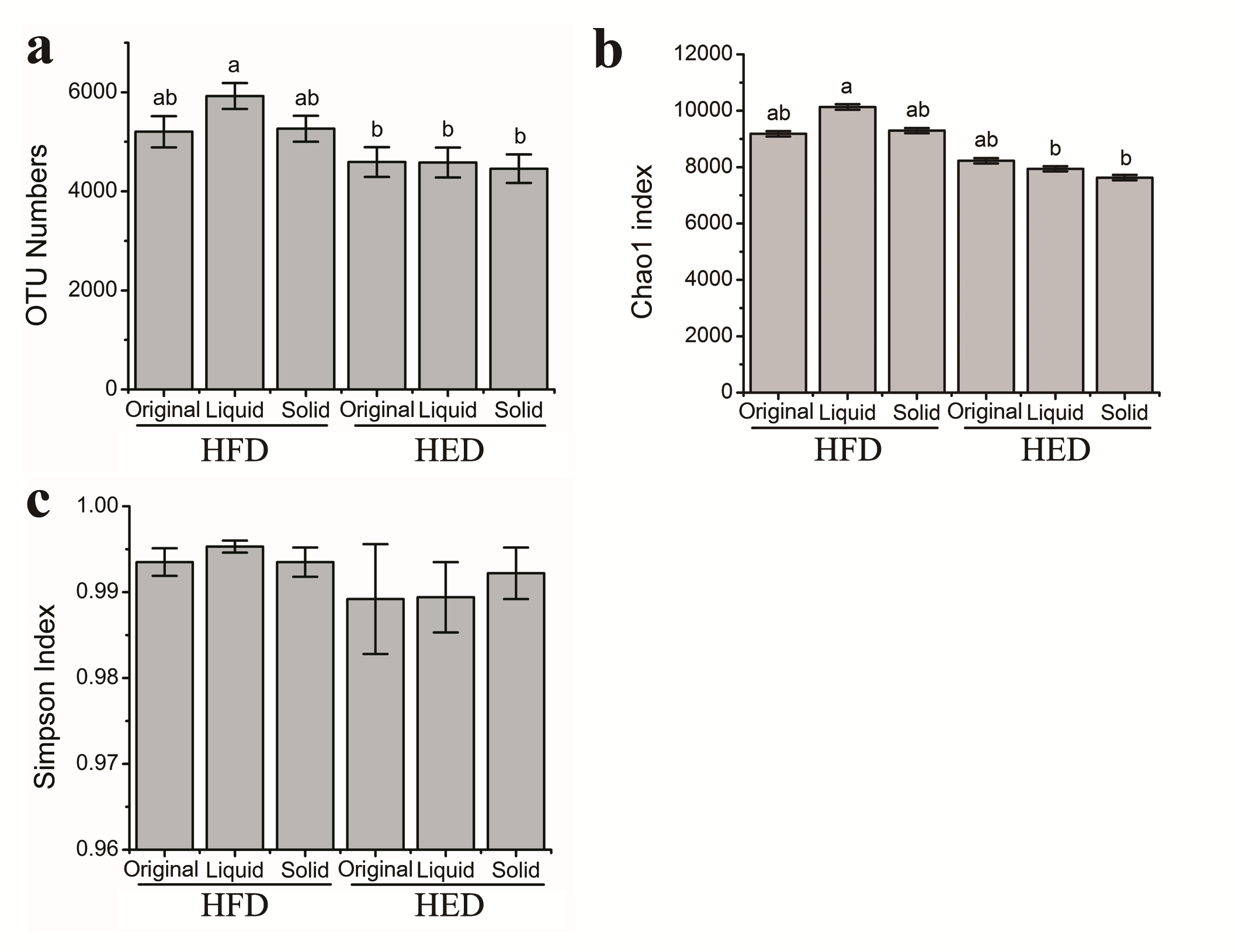


Figure S1


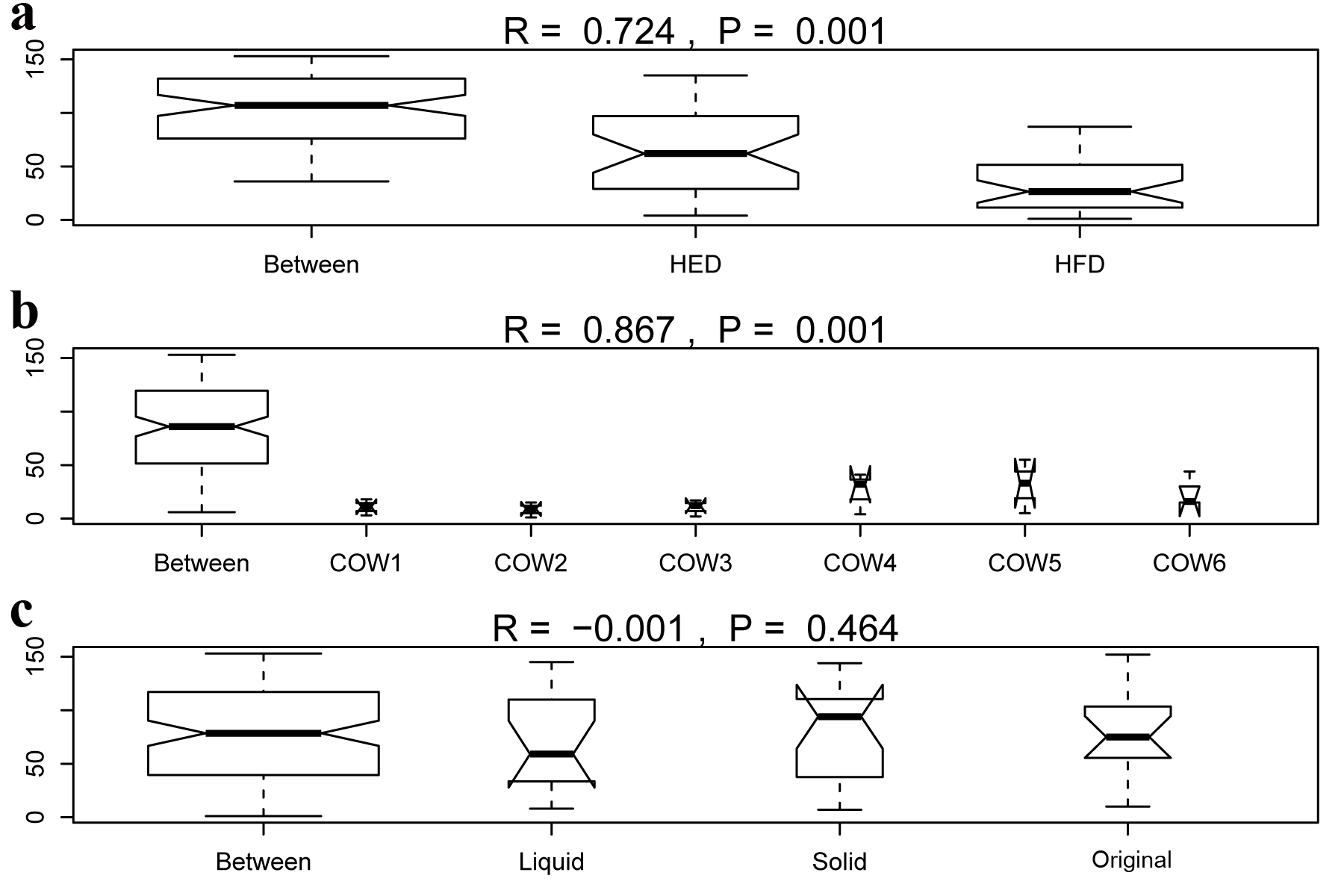


Figure S2


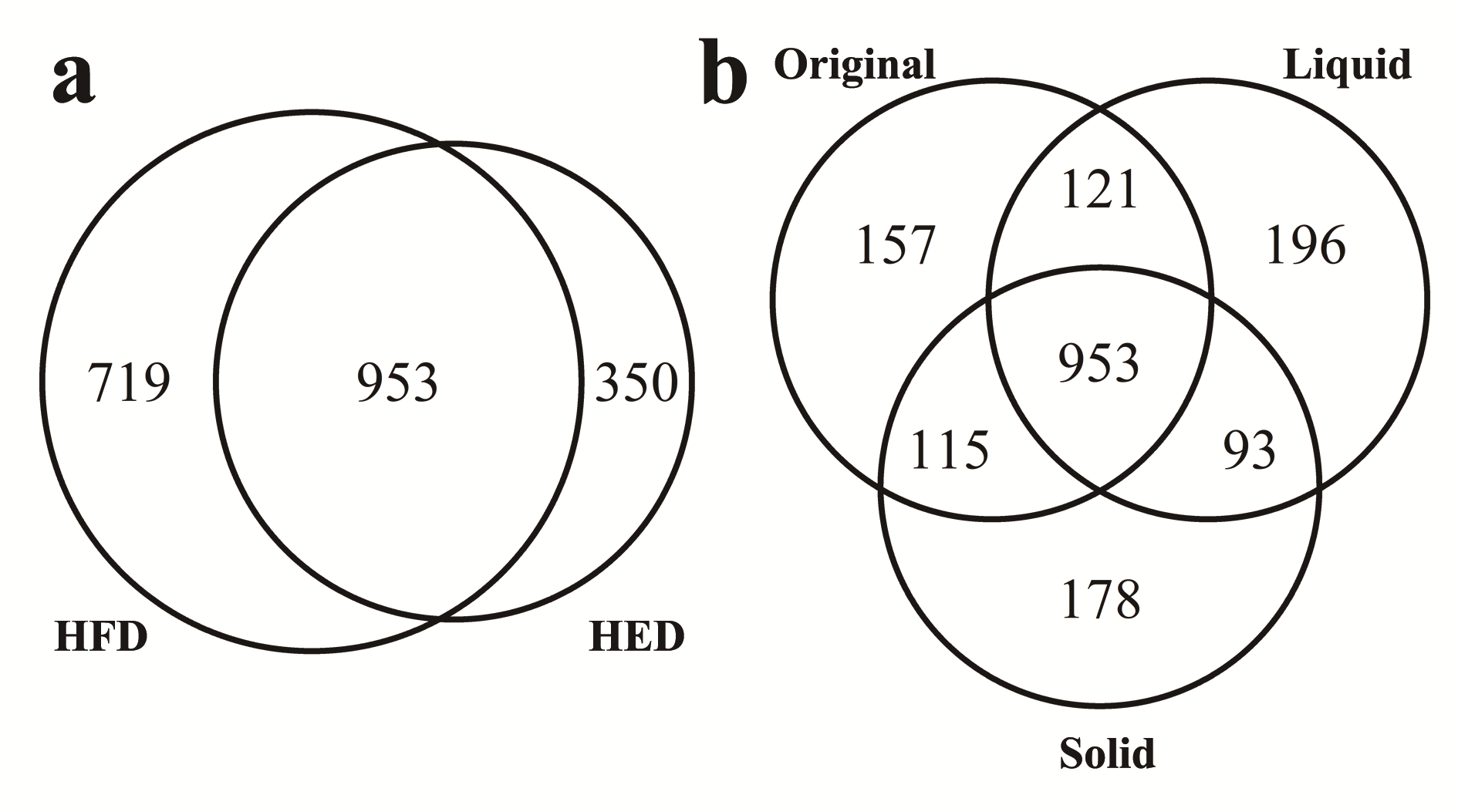


Figure S3


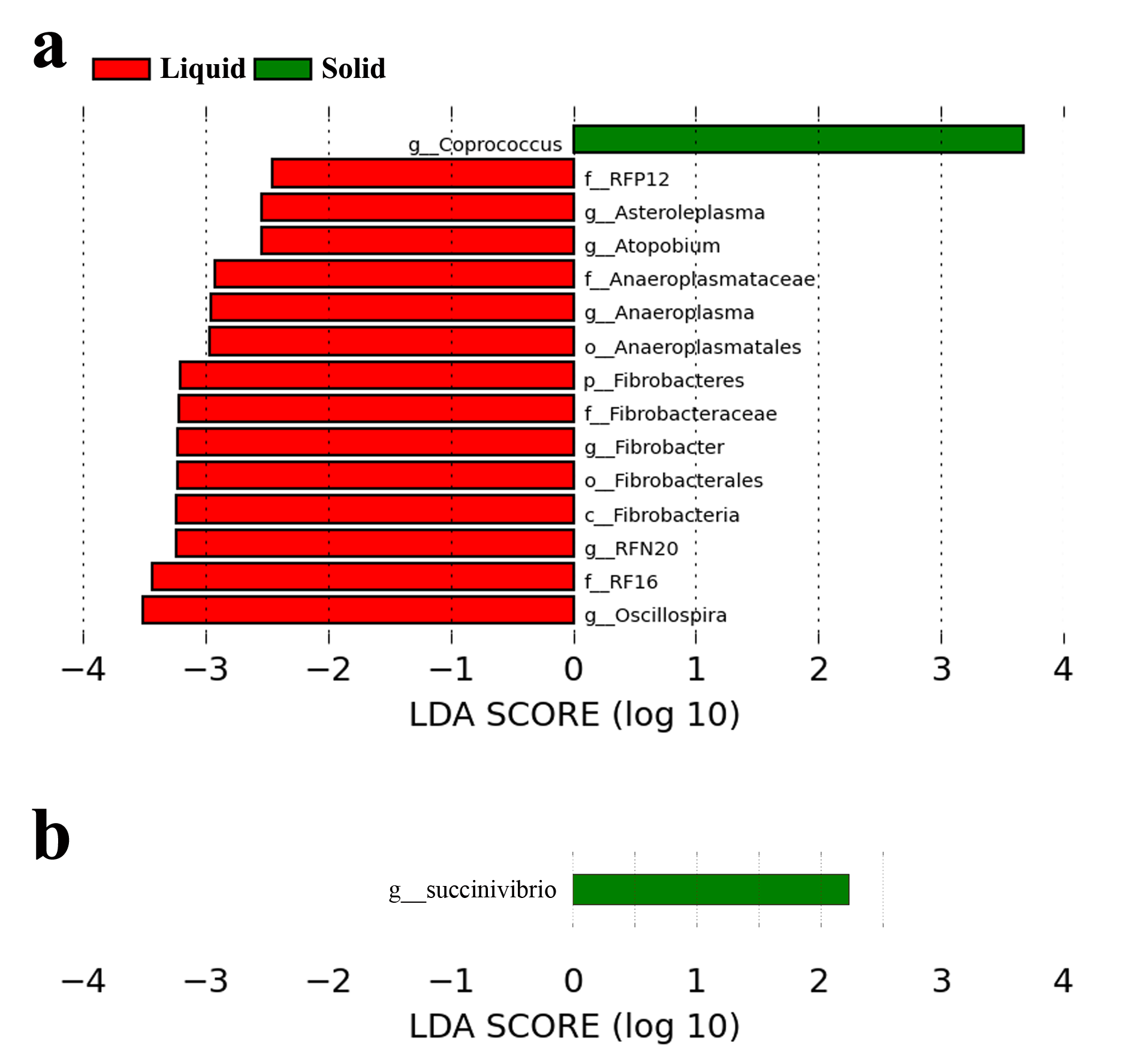


Figure S4


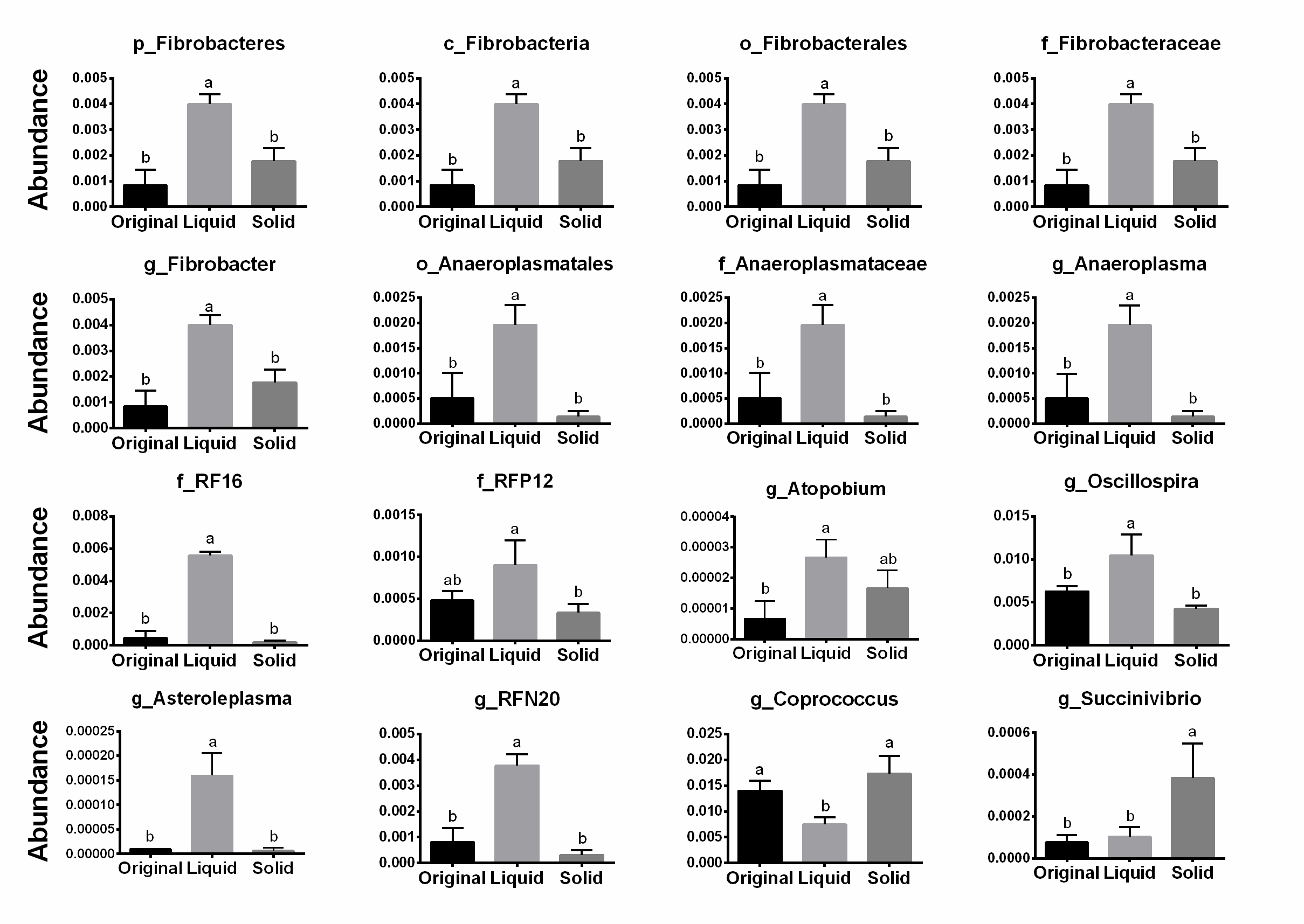


Figure S5


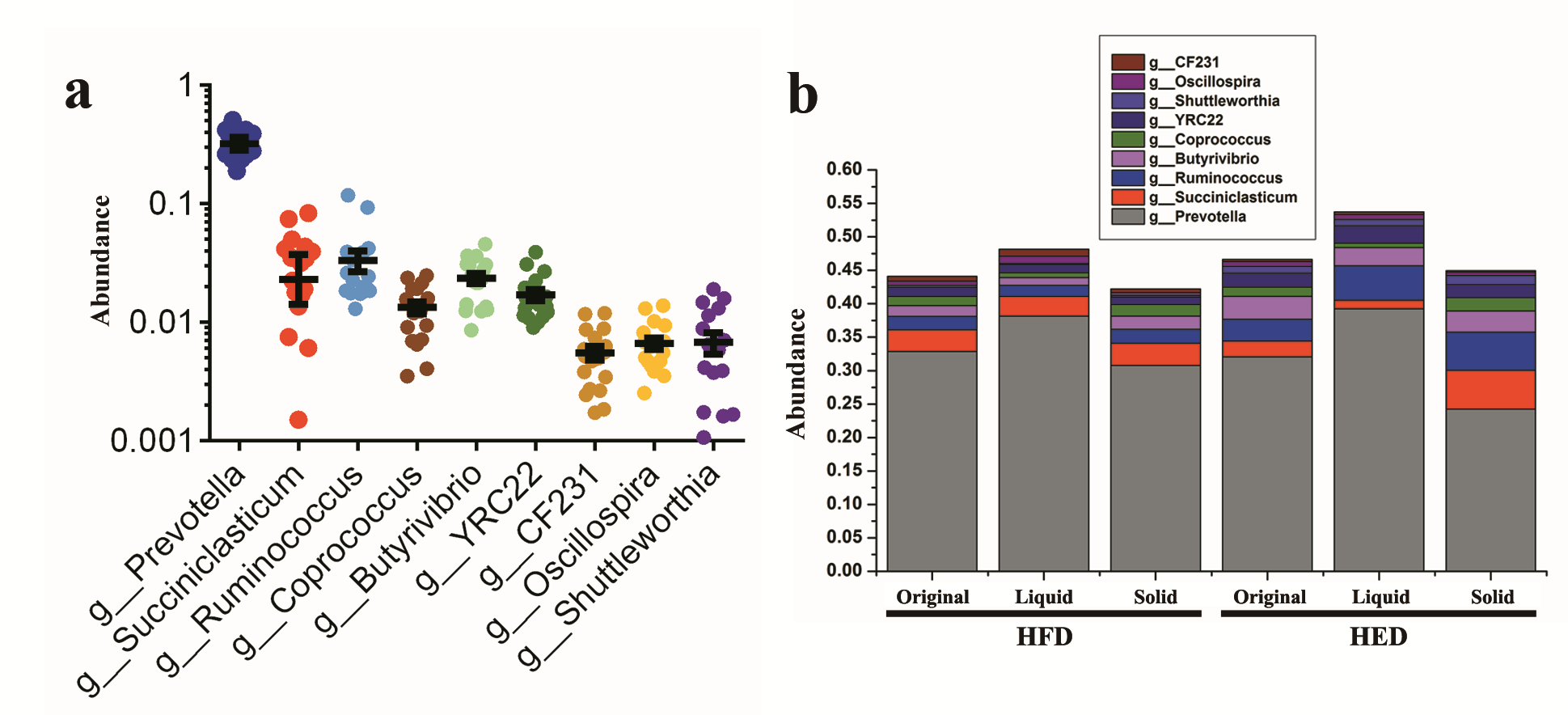


Figure S6
